# Dynamic input-dependent encoding of individual basal ganglia neurons

**DOI:** 10.1038/s41598-020-62750-0

**Published:** 2020-04-02

**Authors:** Ayala Matzner, Lilach Gorodetski, Alon Korngreen, Izhar Bar-Gad

**Affiliations:** 1 The Leslie & Susan Goldschmied (Gonda) Multidisciplinary Brain Research Center, Bar-Ilan University, Ramat-Gan, Israel; 2 Goodman Faculty of life sciences, Bar-Ilan University, Ramat-Gan, Israel

**Keywords:** Neuroscience, Computational models, Computational neuroscience, Neural encoding

## Abstract

Computational models are crucial to studying the encoding of individual neurons. Static models are composed of a fixed set of parameters, thus resulting in static encoding properties that do not change under different inputs. Here, we challenge this basic concept which underlies these models. Using generalized linear models, we quantify the encoding and information processing properties of basal ganglia neurons recorded *in-vitro*. These properties are highly sensitive to the internal state of the neuron due to factors such as dependency on the baseline firing rate. Verification of these experimental results with simulations provides insights into the mechanisms underlying this input-dependent encoding. Thus, static models, which are not context dependent, represent only part of the neuronal encoding capabilities, and are not sufficient to represent the dynamics of a neuron over varying inputs. Input-dependent encoding is crucial for expanding our understanding of neuronal behavior in health and disease and underscores the need for a new generation of dynamic neuronal models.

## Introduction

Characterizing the computational properties of individual neurons is crucial for understanding their contribution to normal brain function and its breakdown during different pathologies. The basic unit of the computation the neuron performs on its inputs into action potentials is a transformation typically termed “neural encoding”. An essential set of tools for studying this encoding are computational models, which are a simplified representation of the neurons. By modeling the relationship between stimuli (e.g., visual scenes) and the spiking responses of neurons, computational models aim to account for the mechanisms underlying neuronal computation^1,2^. Modeling this relationship serves to predict neuronal responses under different conditions, and facilitate the study of the fundamental properties of neuronal encoding in the normal state as well as changes in different pathological states^3,4^.

A variety of computational models have been put forward to approximate the activity of experimentally recorded neurons at different levels of abstraction while maintaining biological plausibility^5–7^. This abstraction level ranges from simple models, such as the integrate-and-fire model, to biologically detailed models. The choice of abstraction level depends on the particular goal of the model, and the question addressed by the model. Computational models can be divided into deterministic and statistical models. Deterministic models include most biophysical models, with a broad range of complexity, and typically map the model’s parameters to biophysical properties. In contrast to deterministic models in which the same input will always result in the same output, statistical models reflect the stochastic response of neurons to similar repetitive input. Statistical models do not typically utilize parameters which have a biophysical meaning but instead represent conceptual properties related to the neuron’s computation^1^.

A common statistical model is the generalized linear model (GLM), a generalization of linear regression which relates a linear model to observations from any probability model in the exponential family via a non-linear linking function. The GLM framework has been adapted to point process data, which are often referred to as point process GLM (PP-GLM)^8,9^, and has become a standard way to analyze and model spike train data^10–12^. Here, for simplicity, we use the general term GLM to refer to PP-GLM. The GLM typically incorporates a linear stimulus filter which accounts for stimulus encoding, a spike history function which captures effects such as refractory periods, bursting and other non-Poisson features of spike train statistics, and a bias term which reflects tonic firing. These filters are combined to generate an input to a Poissonian neuron. GLMs have been shown to outperform other models, both for predicting spike trains (neural encoding) and for performing stimulus reconstruction (neural decoding)^12,13^. GLMs have been applied to neurons in sensory systems, where they associate known external stimulus features and spiking output^2,14,15^, and in the context of neurons *in vitro*, where the input is either injected somatic current or synaptic stimulation^13,16,17^.

GLMs belong to the family of static models which includes models utilizing multiple levels of abstraction. These models are characterized by a set of parameters which account for the neuronal computational properties regardless of the inputs, thus forming context independent models^18–20^. This static structure enables the model to be a robust representation of the experimentally recorded neuron. This allows the model to represent the invariant encoding properties of the neuron, which can consequently be used to study the computational properties of individual neurons or networks consisting of these neurons. Here we examine this hypothesis by analyzing computed GLMs under different input conditions. We focus on basal ganglia (BG) neurons since their firing rates range broadly on both the single neuron level, as well as the population level. We show how the parameters of the computed GLM of the same neuron change with the input statistics and with the firing rate of the neuron, leading to changes in the basic computational properties and information capacity of these neurons under different conditions. Thus, neurons display dynamic, input dependent enhanced computational capabilities beyond a single, static, context independent, computational model.

## Results

### Modeling cellular integration of input using GLMs

**Figure 1:**
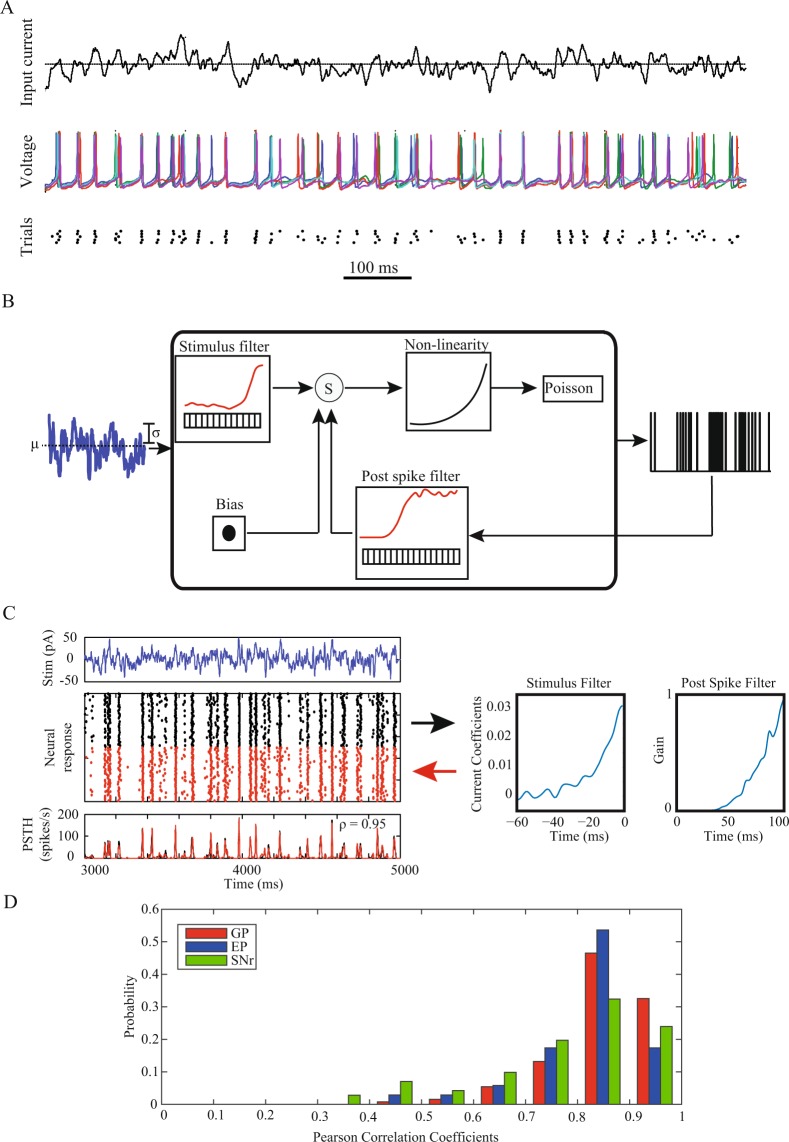
GLM as a tool for modeling cellular integration of input. (**A**) Frozen noise injected into an EP neuron over multiple trials (top). Example of 5 intra-cellular potentials (different colors) of the neuron during repeated injections of the frozen noise (middle). Raster display of the neuronal spiking over the same trials (bottom). (**B**) Schematic structure of the GLM: Input passing through the stimulus filter is summed with the output spikes serving as the input to the post spike filter and a constant bias term and passing through a non-linear function (exponent) to generate an input to a Poissonian spike generator. (**C**) Left - The GLM spiking responses to a novel fluctuating current stimulus. From top to bottom: input stimulus, raster plots of spiking activity for repeated stimulus presentations over 50 trials of the experimental neuron (black) and the GLM reconstruction (red), PSTHs of the responses of the real neuron (black), and the GLM response (red). The prediction accuracy is assessed using the PCC between the experimental and model PSTHs. Right - The derived stimulus filter and post spike filter of the fitted GLM. The post spike filters are shown in their exponentiated form. (**D**) Histogram of the Pearson Correlation Coefficients (ρ) between the PSTH of the neuronal responses to the frozen noise and the PSTH of the model response to the same noise in the GP (red), EP (blue) and SNr (green).

We recorded the activity of globus pallidus (GP) (n = 57), entopeduncular nucleus (EP) (n = 29) and substantia nigra pars reticulata (SNr) (n = 32) neurons during *in-vitro* whole-cell recordings following the blockade of GABAergic and glutamatergic transmission. We injected a “frozen noise” current, which was generated by convolving white noise with an alpha function, into the soma of the neurons repeatedly over multiple trials (30–50 trials) separated by periods without noise injection (see methods). This current approximates natural inputs received by the neurons *in vivo*^21^. The spiking activity was identified from the recorded intracellular potential of the neurons and was used to generate a point process (Fig. 1A). The neuronal encoding of the stimulation was estimated using a generalized linear model (GLM) utilizing a non-linear function (exponent) over the linear filters of the incoming stimulus, the post spike filter and a constant bias term (Fig. 1B, see methods). The parameters of the GLM were fitted to the neuronal responses to maximize the fitting to the training set (using the initial 80% of the stimulation period). The resulting GLM was then used to generate simulated spike trains, and the smoothed PSTH was compared to that of the experimental neuron using the test set (last 20% of the stimulation period) and scored using Pearson’s correlation coefficient (PCC). The GLM accurately reproduced the responses of the recorded cell, and the resulting firing rate provided an estimation of the *in-vitro* recorded firing rate (Fig. 1C). Overall, the GLM representation of the neurons demonstrated highly correlated responses to the experimental neuronal responses (Median PCC: GP: 0.86, EP: 0.83, SNr: 0.83) (Fig. 1D).

### The parameters of GLMs are dependent on the input statistics

**Figure 2:**
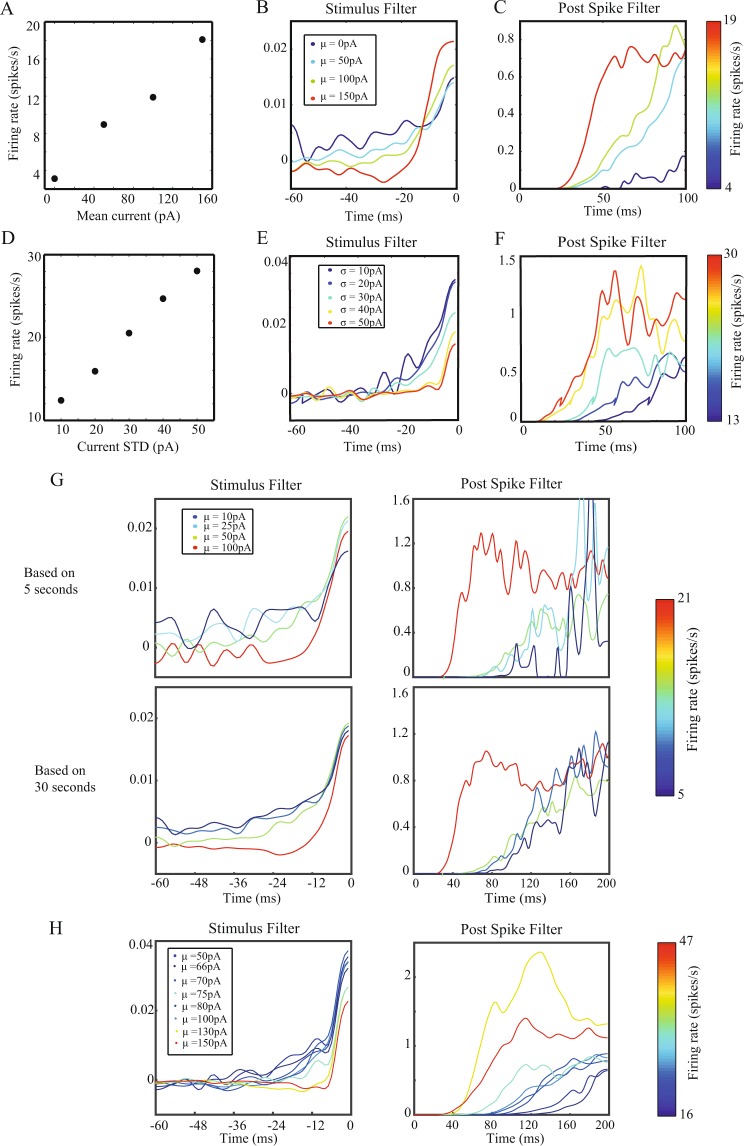
The parameters of GLMs are dependent on the input statistics. (**A–C**) The effect of the mean current of the injected stimulus on the fitted GLM filters. (**A**) The relationship between the mean injected current and the resulting firing rate. (**B**) Stimulus filters of a GP cell for rates of 4–19 Hz varied by increasing the input DC. (**C**) The corresponding post spike filters. (**D–F**) The effect of changing the variance of the stimulus while keeping the mean DC constant (subfigures correspond to A–C). (**G**) The fitted GLM filters of short (5 second) (top) and long (30 second) stimulation periods of the same neuron over different firing rates. (**H**) The fitted stimulus filters and post spike filters using a GLM that includes an additional firing rate filter over the previous 500 ms of the same neuron over different firing rates.

We next examined the “invariance assumption” underlying most studies which use GLMs^2,12,13,22^. This assumption is that the neuron’s fitted GLM parameters are invariant to the input statistics, such that different inputs to the same neuron will result in the generation of similar GLMs. The essence of this assumption implies that the neuron’s computational properties are stable across input conditions. We changed the mean and variance of the frozen noise injected to the neuron and compared the resulting GLMs. We varied the constant direct currents (DCs) added to the fluctuating noise, and this affected the mean firing rate (Fig. 2A), and both the fitted stimulus filter (Fig. 2B) and the post spike filter (Fig. 2C) of the generated GLMs, such that different filters were generated based on the baseline current. In the example neuron, the increase in the rate correlated with an increase in the peak magnitude of the stimulus filter and a shortening of the inhibitory period (filter values ≪1) of the post spike filter. An increase in the variance (between 10 to 50 pA) of the frozen noise while maintaining the same baseline DC led to similar increases in the firing rate (Fig. 2D), and variations in both the stimulus filter (Fig. 2E) and the post spike filter (Fig. 2F). In the example neuron, the increase in variance correlated with a decrease in peak magnitude of the stimulus filter and a shortening of the inhibitory period of the post spike filter. In order to rule out the possibility that these results were due to the relatively short stimulation durations used for generating the GLMS leading to a limited number of spikes, we recorded neuronal activity while injecting a long (30 second) current of frozen noise. We then generated GLMs by first taking only the first 5 seconds of the recording (Fig. 2G, top), and then using the whole recording duration (Fig. 2G, bottom). The longer recordings only slightly improved the divergence of the GLM parameters over different baseline firing rates and resulted primarily in a smoother representation of the GLM parameters. Using filters of longer durations did not change the results as well, as the filters converge to 0 for longer latencies. Thus, using short stimulation periods sufficed to generate GLM parameters, and was not the cause of their variations across different input properties. Adding a firing rate filter which captured the previous mean firing rate of the neuron produced similar results, where the shape of both the stimulus filters and the post spike filters varied with the change in the firing rate of the neuron (Fig. 2H).

### GLMs fitted over different input statistics are not interchangeable

**Figure 3:**
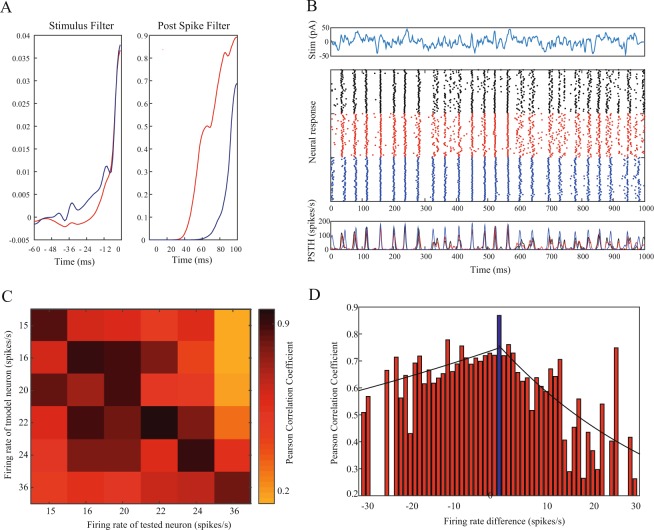
GLMs fitted over different input statistics are not interchangeable. (**A**) Two GLMs (stimulus filter and post spike filter) of the same cell, generated using different mean DCs (100 and 66) added to the same frozen noise stimulus, which result in different firing rates: red – 24 Hz, blue – 15 Hz (**B**) Example predictions of the two GLMs in *A* to a novel stimulus with the higher DC. Top - the input stimulus. Middle – the responses of the experimental neuron (black) and the model neurons using the GLM calculated using the higher DC (red), and the lower DC (blue). Both model neurons’ mean firing rate is similar (24 Hz) to the rate of the real neuron, achieved by applying the proper constant DC. Bottom – the corresponding PSTHs. The correlation between the experimental PSTH and the model PSTH of the higher DC (red) is 0.89, and the lower DC (blue) is 0.6. (**C**) Generalization of GLMs calculated using different firing rates of the same cell leads to reduced accuracy. The GLM generated using a specific DC, which resulted in a specific firing rate, was then used as a model neuron to generate responses to a novel stimulus with different constant DCs. The model PSTHs were tested against the real responses of the cell using the correlation measure as in *B*. (**D**) Same as *c*, but for the whole population. GLMs were generated using a specific firing rate of a neuron, and their prediction accuracy was assessed (the mean of the whole population represented as a firing rate difference of zero, blue bar). The GLM was then used as a model neuron to predict responses of other firing rates of the neuron (represented as ± firing rate difference); the PSTHs correlations were measured, and their mean for each firing rate difference is presented. Significance is measured in relation to the mean PCCs of the original GLMs (firing rate difference of zero), and only significant mean correlations are presented. Black solid lines represent the exponential fitting functions for the positive (R^2^ = 0.48, p < 0.001) and negative (R^2^ = 0.32, p < 0.01) firing rate differences.

The dependence of the GLM fitted parameters on the properties of the input is not merely a case of different parameter combinations that lead to similar model performance. The different GLMs are not interchangeable; i.e., when a model neuron receives the same input, with two different DC levels that lead to two different baseline firing rates, the two GLMs generated using each condition do not only look different (Fig. 3A), but also generate different predicted spike trains and form different PSTHs (Fig. 3B). In the example, the spike trains generated from the GLM that was fitted with the different baseline current were less sparse and more accurate in time than the experimental spike trains, as well as those generated with the GLM created with the same current baseline as the test data. Therefore, when comparing the predicted PSTH to the real PSTH of the test data, only the GLM that was fitted using the same recording conditions as the test data generated responses that were highly correlated to the responses of the experimental neuron to the incoming current. Using the GLMs of the same neuron that were generated from recordings with different current properties led to responses that differed from the real responses (Fig. 3C). We tested 42 neurons recorded over multiple sessions, where in each session, we injected the same noise with a different baseline current. The inability to generalize GLMs from different firing rates of the same neuron was a prominent feature and was expressed in most of the sessions pairs as a reduced correlation between the responses (Fig. 3D). This trend of reduced generalization was smaller when the firing rate of the original neuron was higher than the tested neuron, which indicates that some generalization exists in this case.

### GLM parameters are dependent on the firing rate

**Figure 4:**
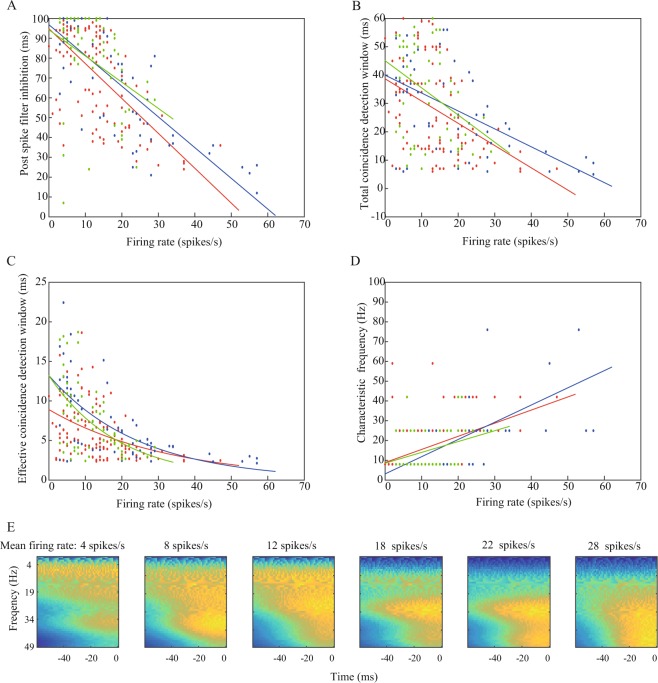
GLM parameters as a function of the firing rate. The dependence of GLM parameters of GP (red), EP (blue) and SNr (green) neurons on the firing rate. In all cases, except *C*, the solid lines represent the linear regression functions. (**A**) Post spike filter inhibition, expressed as the time when the filter reaches its half height, as a function of the firing rate (GP: R^2^ = 0.4, p < 0.001, EP: R^2^ = 0.65, p < 0.001, SNr: R^2^ = 0.14, p < 0.01). (**B**) Stimulus filter total coincidence detection window as function of the firing rate (GP: R^2^ = 0.17, p < 0.001, EP: R^2^ = 0.32, p < 0.001, SNr: R^2^ = 0.15, p < 0.01). (**C**) Stimulus filter effective coincidence detection window as function of the firing rate. Solid lines represent the exponential fitting function (GP: R^2^ = 0.19, p < 0.001, EP: R^2^ = 0.43, p < 0.001, SNr: R^2^ = 0.28, p < 0.001). (**D**) Stimulus filter characteristic frequency computed from the Fourier transform of the filter, as function of the firing rate (GP: R^2^ = 0.23, p < 0.001, EP: R^2^ = 0.49, p < 0.001, SNr: R^2^ = 0.13, p < 0.01). (**E**) Time-frequency representation of the GLMs of SNr neurons. The representation is derived from a Morlet wavelet transform of the mean stimulus filters of sessions with a similar firing rate (±2 Hz).

These results suggest that neurons’ computational properties can differ as a function of input properties. To quantify the systematic changes in these properties, we analyzed all the GLMs that we generated from the experimental neuron recordings. In some of these neurons, we recorded activity during multiple sessions while changing the DC, which yielded a dataset with diverse firing rates. We analyzed 119 sessions from GP neurons, 61 sessions from EP neurons, and 51 sessions from SNr neurons. In each of the nuclei, the GLM parameters were highly dependent on the firing rate of the neuron. The post spike filter exhibited a strong negative correlation between the duration of inhibition and the firing rate (Fig. 4A). To analyze the stimulus filter, we calculated the total coincidence detection window; i.e., the duration of the positive part of the filter where the neuron sums the arriving inputs, and the effective coincidence detection window which measures the effective duration in the positive part, when the shape of the stimulus filter displays a sharp decay to zero. Both the total and the effective coincidence detection windows were significantly negatively correlated with the firing rates in all the nuclei, such that higher firing rates narrowed the coincidence detection windows (Fig. 4B,C). We further analyzed the spectral selectivity of the stimulus filters by looking at the characteristic frequency of the Fourier transform of the filter (Fig. 4D). Similar to the coincidence detection windows results, the spectral results presented a positive correlation between the firing rates and the main frequency, and indicated that neurons that fired at low rates acted as low pass filters, whereas at higher firing rates the same neurons tended to display a high pass filtering activity. This phenomenon could also be seen at the single neuron level using wavelet analysis: when recording the same neuron over different firing rates, the neuron’s stimulus filters changed their spectral selectivity depending on the firing rates (Fig. 4E).

### Verification of experimental results using model neurons

**Figure 5:**
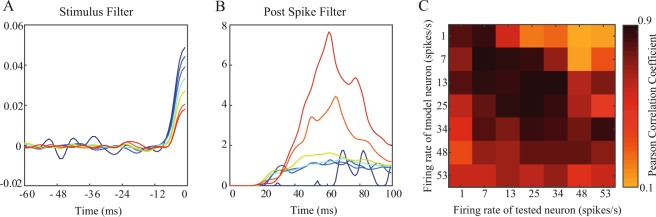
Model neurons demonstrate similar results. (**A–C**) the effect of firing rate on GLMs generated from detailed biophysical neuronal model. (**A**) Divergence of stimulus filters of a LIF cell model neuron for varying rates (**B**) The corresponding post spike filters. (**C**) Generalization of the GLMs calculated using different firing rates of the same cell leads to reduced accuracy.

We tested the results we collected experimentally by comparing them to simulations using a detailed biophysical neuronal model (implemented with the NEURON modeling environment). This served to verify a wide range of input parameters (mean and variance) and specifically long-term activity. The GLM representation of the spike trains generated by the detailed biophysical neuronal model exhibited the same dependence on the firing rates, such that the shape of both the stimulus filters and the post spike filters were dependent on the baseline firing rate (Fig. 5A,B). Furthermore, as in the experimental neurons, the filters were not interchangeable (Fig. 5C).

### The fidelity and accuracy of the neuronal transformation depends on the firing rate

**Figure 6:**
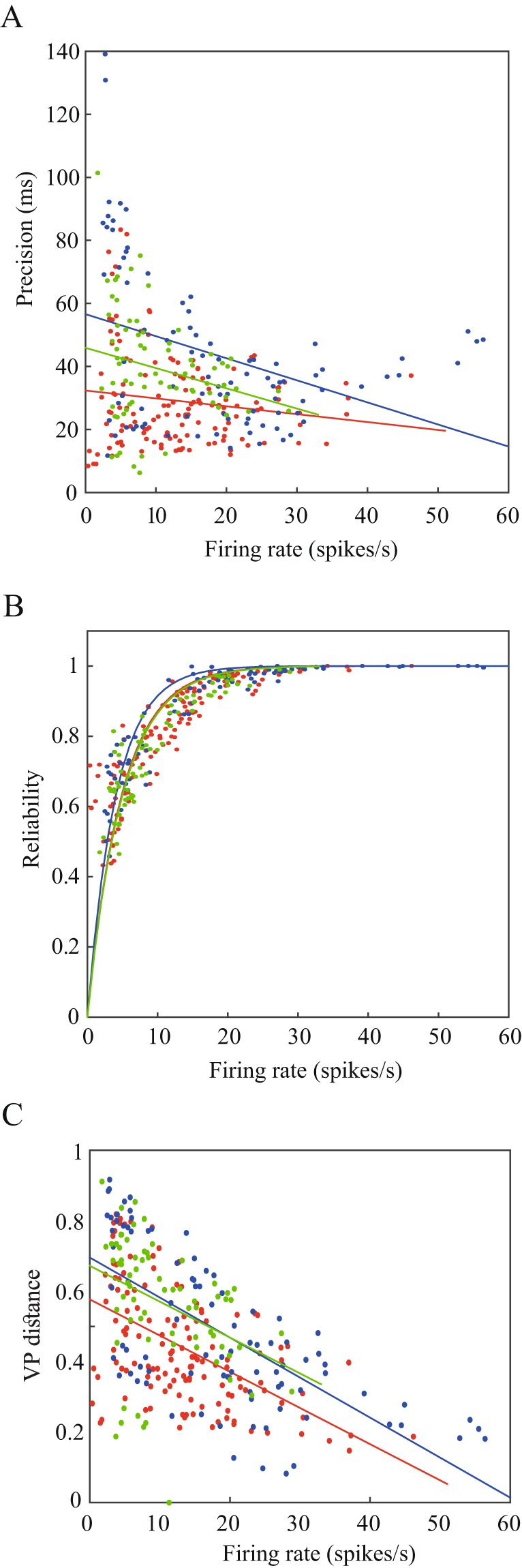
Dependence of the fidelity and accuracy of the neuronal transformation on its firing rate. Results shown for neurons from the GP (red), EP (blue) and SNr (green). (**A**) Precision of the neuronal responses to multiple injections (30–50) of the same input, as a function of the firing rate. The precision is defined as the mean widths of the events in the smoothed PSTH of each session. Solid lines represent the linear fitting function (GP: R^2^ = 0.02, p = 0.08, EP: R^2^ = 0.14, p < 0.05, SNr: R^2^ = 0.06, p < 0.05). (**B**) Reliability estimation of neuronal responses to repeated injections of the same noise, calculated as the fraction of spikes that occurred in PSTH events. Solid line represents the exponential fitting function (GP: R^2^ = 0.43, p < 0.001, EP: R^2^ = 0.78, p < 0.001, SNr: R^2^ = 0.66, p < 0.001). (**C**) Victor-Purpura distance (q = 0.15) as a function of the firing rate. Each dot represents the mean Victor-Purpura distance between all pairs of spike train trials recorded in a single session, as a function of the neuron firing rate in that session. The solid lines represent the linear fitting function in each of the nuclei (GP: R^2^ = 0.29, p < 0.001, EP: R^2^ = 0.47, p < 0.001, SNr: R^2^ = 0.14, p < 0.01).

Next, we examined whether the changes in the computational properties of the experimental neurons during different firing rates affected the information transmission of those neurons. A key property of information processing is the maintenance of reliability; i.e., a particular stimulus will lead to similar neuronal activity each time it is presented. Another feature of information processing is the temporal precision of the responses, which is the timescale of the jitter between responses on different trials to the same input. The precision and the reliability were derived from the smoothed PSTH of the responses of the neuron to multiple injections of the same frozen noise, thus representing the instantaneous firing rate. We then identified elevations in the PSTH, which we termed events. The temporal precision was defined as the mean widths of the event, and the reliability was defined as the fraction of spikes that occurred within the event. The precision of single sessions was negatively correlated with the firing rate in all the nuclei (Fig. 6A), and the reliability had a significant asymptotic relation with the firing rate (Fig. 6B). The combination of the dependence of these features on the firing rate was expressed as the distance between pairs of spike trains from the same session where pairs of spike trains with higher firing rates are expected to be more similar. We computed the distance using the Victor-Purpura metric (q = 0.15)^23,24^. In order to rule out the effect of the firing rate, we used a normalized version of this distance^25^. For each session, the distance was defined as the mean distance between all pairs of spike trains within the session. As expected, in all the nuclei, there was a significant negative relationship between spike train distance and firing rate (Fig. 6C).

### Similarity of neuronal encoding of pairs of neurons within and between nuclei

**Figure 7:**
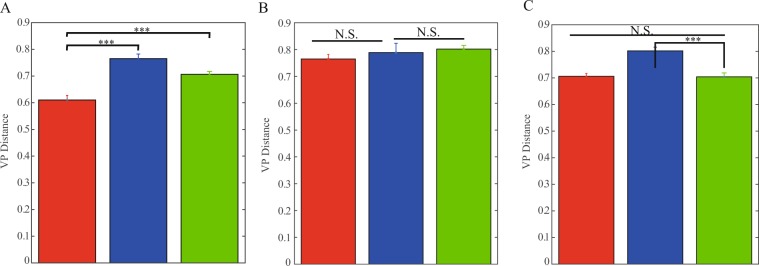
Similarity between neuronal encoding of pairs of neurons within and between nuclei. (**A-C**) Mean Victor-Purpura distance between pairs of spike trains from different neurons with the same baseline firing rate, during injection of similar currents. The comparison is between the distances of pairs within the same nucleus to distances of pairs in different nuclei. (**A**) Red: GP-GP pairs (±SEM, n = 89, p < 0.001, Wilcoxon rank sum test), blue: GP-EP pairs (n = 73, p < 0.001), green: GP-SNr pairs (n = 119, p < 0.001). (**B**) Red: EP-GP pairs (±SEM, n = 73, N.S., Wilcoxon rank sum test), blue: EP-EP pairs (n = 24), green: EP-SNr pairs (n = 47, N.S.). (**C**) red: SNr-GP pairs (±SEM, n = 119, N.S., Wilcoxon rank sum test), blue: SNr-EP pairs (n = 47, p < 0.00), green: SNr-SNr pairs (n = 43, p < 0.001).

Finally, we examined the relative effect of the firing rate of a neuron relative to other factors that determine neuronal encoding properties. We analyzed the similarity between neuronal responses of different neurons, within and between nuclei that fired at the same baseline firing rate and received similar input. The assumption was that responses of neurons within the same nucleus would be more similar to each other than to those of different nuclei, as a result of the common morphology and biophysical features of neurons within the same nucleus. If the responses of neurons in different nuclei are similar to responses of neurons in the same nucleus, this would suggest that the firing rate of the neuron influences its computation more than its biological properties. We applied the Victor-Purpura distance to characterize the similarity and dissimilarity of neuronal responses of pairs of neurons receiving the same input noise and having the same baseline firing rates during the recording. Our results showed that in some of the cases, the neuronal responses of pairs of neurons within the same nucleus were as similar as pairs of neuron between nuclei (Fig. 7). This may indicate that the firing rate of the neuron plays a significant role in the computation and the encoding properties of neurons.

## Discussion

Context independent modeling studies assume that a fixed set of parameters can model a neuron and that this model is invariant to input statistics. In this study, we challenged this assumption, using PP-GLMs as a representative model for this type of statistical models. We fitted GLMs to the *in-vitro* whole-cell responses of BG neurons to repeated stimulation. In order for our results to be generalized to *in vivo* neurons, the injected current should simulate the *in vivo* environment, i.e., inputs from active pre synaptic neurons. Thus, we injected currents that mimic those inputs received *in vivo*. The input statistics were varied across sessions, influencing the baseline firing rate of the neurons, and forming their internal state. The GLMs accurately reproduced the responses of the experimentally recorded cells in single sessions. However, these static models were not able to reproduce the neuronal spiking activity in different sessions and different states of the neurons. Furthermore, the parameters of the GLMs were found to be highly sensitive to the firing rate of the neurons.

Using the GLMs, we quantified the encoding of individual neurons and demonstrated how various encoding properties are dependent on the baseline firing rate. We further demonstrated that the information processing properties of single neurons vary with their firing rates. We found similar results for input dependent computation in detailed and simplified biophysical models as apparent in the experimental dataset. This indicates that the input statistics have a major influence on the computation properties of neurons, such that there is no single “real” static encoding function of a neuron, but rather an input dependent encoding scheme. This study combined modeling and experimental approaches to better understand the encoding properties of individual neurons. In terms of modeling, we used GLMs, which incorporate a linear stimulus filter, a spike history filter, and a bias term. The stimulus filter represents computational properties that are related to the input to the neuron, the spike history filter represents the influence of previous spikes on the current response, and the bias term reflects the tonic firing of the neuron. Together, these filters are commonly assumed to represent the computational properties of the neuron to a large extent. The simplicity of the GLM allows for direct interpretation of the encoding properties of the neuron from the model parameters. Using the GLMs, we demonstrated how static models are insufficient to computationally describe single neurons since computational properties change with the input statistics. In order to rule out the possibility that simply adding complexity to the model would resolve the input dependent computation changes illustrated by the GLMs, we tested our results using an extension of the GLM with a firing rate filter. However, this model did not qualitatively change the overall results, thus hinting that merely adding more complexity to the model cannot overcome this limitation of the static model. Specifically, these changes fail to produce a model which is invariant for a wide range of inputs, because this phenomenon is a property of the neuron and not a side effect of a specific model. It is also not a property derived from the duration of the recording session since models fitted on short trials exhibited similar properties to those fitted on much longer trials. Thus, model parameters that are fitted with a specific input are unfit for reproducing the responses of the same neuron to other inputs with different statistics. Instead, these myriads of static models provide valuable qualitative insights into the wide range of possible encodings by single neurons.

These findings are critical for modeling neurons and investigating their computational principles. The firing rates of real neurons typically fluctuate over multiple time scales and over different behavioral states^26,27^. The resulting computation during these different states is not static but rather changes as a function of conditions. Thus, in order to capture the variety of the neuron’s computational properties, experiment must include different states of the neuron, instead of recording the neuron in a single state. Modeling and interpreting the encoding of a neuron in one state might not be pertinent to other states for the same neuron. Previous studies have attempted to overcome these limitations by including the information of the instantaneous membrane potential^28^. These, for example, include models which utilize the state space of the voltage^29^, and models that incorporate the non-linear dynamics of the firing rate threshold into the model^19,30,31^. Variations in spike threshold might affect neuronal computation mechanisms; however, using model neurons, our results suggest that the mechanisms underlying these input dependent computations are more fundamental and complex. Here, input dependent computation was demonstrated in detailed biophysical neurons. This links the underlying mechanisms to some complex mechanisms, which are characteristics of detailed biophysical models. These may include voltage dependent active membrane properties, such as activation of slow potassium currents, sodium inactivation, afterhyperpolarization currents, and changes in spike generation mechanisms due to various voltage dependent changes.

The computation of single neurons has been extensively investigated, and in this framework, different inputs are already known to produce varying firing rates and varying responses in single neurons. In some neural systems (e.g. motor control, visual system), the relationship between stimuli and neural responses has been studied in depth^32^. The computational properties in these conditions have been examined in part in the context of gain modulation; i.e., the change in the input-output function of a single neuron^33–35^. In addition to the somatic computation which we studied, neuronal computation also consists of dendritic computation, which has been shown to be more than simple transmitting devices, in that their nonlinear summation of inputs provide the neuron with a variety of computational functions^36–38^. Here, we explored the influence of the input statistics on the computation inside the soma itself, after receiving and integrating all the inputs from the dendrites. Thus, we blocked all synaptic inputs while injecting current into the soma of the neurons, thus excluding the contribution of the dendrites to this computation, The blockage of synaptic inputs also excluded the influence of neuronal circuits and functional connectivity, both of which are known to change dynamically and influence neuronal computation^39^. Thus, the reconfiguration of computation over different inputs was not influenced by external network connections. Previous studies have shown how neuronal computation depends on the statistical properties of the input^18,40,41^. In this study, the use of GLMs enabled a direct interpretation of the underlying computational principles, since mapping from the GLM filters to computational meaning was straightforward. We showed the dominant dependency of the computation on the internal state of the neuron, such that when the neuron is in a higher firing rate mode, it tends to trend towards functioning as a coincidence detector, while at lower firing rates it has integrator properties. These features were also reflected in the spectral properties of the model filters, where at high firing rates neurons tended to display high pass filtering activity compared to low pass filtering in lower firing rates. Thus, neurons should not be considered as either integrators or coincidence detectors^42^. Our results indicate that these features may vary over states in individual neurons. These properties were consistent across neurons in the different nuclei of the BG. Similar results were observed in the analysis of interneurons in the cortex (Supplementary Fig. 1), which hints that this phenomenon is not limited to BG neurons. These changes also impact information transmission in that neurons tend to transmit information more reliably and precisely at high firing rates than at lower firing rates.

These findings are applicable to any change in neurons’ baseline firing rates, which can occur as a result of changes in behavioral state, and in different pathological conditions that influence the firing rates of the neuron. An example of such a pathology is Parkinson’s disease which leads to firing rate changes throughout the BG^43,44^. Our results imply that this input-dependent computation might contribute to the deficiency in the computational capabilities of these neurons in these different disorders. Subsequent to the changes in the baseline firing rates, the computational properties of the neurons shift to a baseline region which is different from their original baseline, thus influencing their mean computational performance. Finally, we showed how the responses of pairs of neurons in different nuclei were as similar as pairs of neurons within the same nucleus. This finding indicates that the firing rate of the neuron has a considerable influence on its responses, and in some cases is more influential than the morphology and the biological features of the neuron.

Overall, our results suggest that neurons have a repertoire of computational properties which are influenced by the input properties and the baseline firing rate of the neuron. Moreover, information processing is dynamic and dependent on the state of the network. Thus, before modeling any neuron, scientists must understand the complexity of the physiological environment. Then, novel dynamic models are needed to provide a better understanding of neuronal behavior in health and disease.

## Methods

### *In vitro* slice preparation

Brain slices were obtained from 14 to 21-day-old Wistar rats as previously described^45–48^. Rats were killed by rapid decapitation according to the guidelines of the Bar-Ilan University Animal Welfare Committee. This procedure was approved by the National Committee for Experiments on Laboratory Animals at the Israeli Ministry of Health. The brain was quickly removed and placed in ice-cold artificial cerebrospinal fluid (ACSF) containing (in mM): 2.5 KCl, 125 NaCl, 25 glucose, 1.25 Na_2_HPO_4_, 2 CaCl_2_, 15 NaHCO_3_, 1 MgCl_2_ and 0.5 Na-ascorbate (pH 7.4 with 95% O_2_/5%CO_2_). In all experiments the ASCF contained APV (50 μM), CNQX (15 μM) and GABAzine (20 μM) to block NMDA, AMPA and GABA receptors, respectively. Thick sagittal slices (320–350 μm) were cut on an HM 650 V Slicer (Microm International, Walldorf, Germany) and transferred to a submersion-type chamber where they were maintained for the remainder of the day in ACSF at room temperature. Experiments were carried out at 37 °C, and the recording chamber was constantly perfused with oxygenated ACSF.

### *In vitro* electrophysiology

*In vitro* recordings form GP, EP and SNr neurons were done as previously described^46,48,49^. Individual GP, EP and SNr neurons were visualized by infrared differential interference contrast microscopy using an Olympus BX51WI microscope with a 60x water immersion objective. Electrophysiological recordings were performed in the whole-cell configuration of the patch-clamp technique under visual control using a CCD camera. Recordings were obtained from the soma of GP, EP and SNr neurons using patch pipettes (4–8MΩ) pulled from thick-walled borosilicate glass capillaries (2.0 mm outer diameter, 0.5 mm wall thickness, Hilgenberg, Malsfeld, Germany). The standard pipette solution contained (in mM): 0.5 EGTA, 10 HEPES, 140 K-gluconate, 4 MgATP, 10 NaCl, 5 L-glutathione, 0.05 Spermin, and 0.4 GTP (pH 7.2 with KOH; Sigma, St Louis, MO, USA). Under these conditions, the Nernst equilibrium potential for chloride was calculated to be –69 mV. The reference electrode was an Ag–AgCl pellet placed in the bath. Voltage signals were amplified by an Axopatch-200B amplifier or Axopatch-700B (Axon Instruments, Union City, CA, USA), filtered at 5 kHz and sampled at 10 kHz. The 10-mV liquid junction potential measured under these ionic conditions was not corrected for.

The recordings were conducted while injecting a filtered white noise current stimulus. The noise traces were a 5 second white noise current convolved with an alpha function with a 3 ms rise time, and each stimulus was presented for ~50 repetitions, separated by periods without noise injection. This stimulus is known to produce reliable spiking^13,50^. In some of the sessions we injected the fluctuating current plus a steady state current to induce variable firing rates. The dataset only included recordings displaying stable input resistance, a stable I-F curve, and stable firing patterns across trials throughout the experiment.

### Generalized linear model

A GLM was fitted to each recording session. The models consisted of a temporal stimulus filter ***k***, a post-spike history filter ***h***, and a constant bias term ***b***^13,15^. The stimulus and history filters were represented with 15 and 25 boxcars basis functions of 4 ms, respectively, spaced equally in time. The time-dependent firing rate of each neuron was modeled as *λ(t)* *=* *exp(****k*** ∙ *x* + ***h*** ∙ *r* + ***b****)*, where *x* was the stimulus and *r* was the neuron’s spike train history. The stimulus filter and spike history filter were defined as vectors whereas the bias term was a scalar. Before fitting, the stimuli were down-sampled to 1 KHz and normalized by subtracting the DC component and dividing by the amplitude of the stimulus noise, as in standard procedures^12^. The model was trained using 80% of the stimulus presentations from all the trials, and the validation was done on the remaining 20%. Following maximum likelihood fitting, the GLM converges to a global error minimum^51^. The quality of the fit was assessed by comparing the PSTH of simulated spike trains to that of experimentally recorded spike trains computed from the test data, using Pearson’s correlation coefficient. The PSTHs were smoothed using an 11-ms Gaussian window.

### Detailed biophysical neurons simulations

We simulated detailed biophysical neurons from detailed neuronal model descriptions provided by the Neocortical Microcircuit Collaboration Portal^52,53^. These neuron models were constructed to correspond to experimental cortical neurons from layers 2/3 and layer 5. We used the NEURON simulation environment^54^ to generate voltage responses to current step injections in these models.

## Supplementary information

Supplementary Information.

## Data Availability

The data sets generated during and/or analyzed during the current study are available from the corresponding author on reasonable request.

## Code availability

Data analysis was performed using MATLAB (Mathworks) and python. The code is available from the corresponding author upon request.
