# Supplementary material for: Dynamic input-dependent encoding of individual basal ganglia neurons
Source: Sci Rep. 2020 Apr 2;10:5833. doi: 10.1038/s41598-020-62750-0 (PMC7118110; doi:10.1038/s41598-020-62750-0)
Supplement: Supplementary file 1 — Supplementary Information. [file 41598_2020_62750_MOESM1_ESM.docx]

**Dynamic input-dependent encoding of individual basal ganglia neurons**

Ayala Matzner^1^, Lilach Gorodetski^2^, Alon Korngreen^1,2^ and Izhar Bar-Gad^1,*^

*^1^The Leslie & Susan Goldschmied (Gonda) Multidisciplinary Brain Research Center, Bar-Ilan University, Ramat-Gan, Israel*

*^2^Goodman Faculty of life sciences, Bar-Ilan University, Ramat-Gan, Israel*

* **Correspondence**:

Izhar Bar-Gad

Bar-Ilan University

Gonda Brain Research Center

Ramat-Gan 52900, Israel

Phone: + 972-3-5317141

Fax: + 972-3-5352184

Email: izhar.bar-gad@biu.ac.il


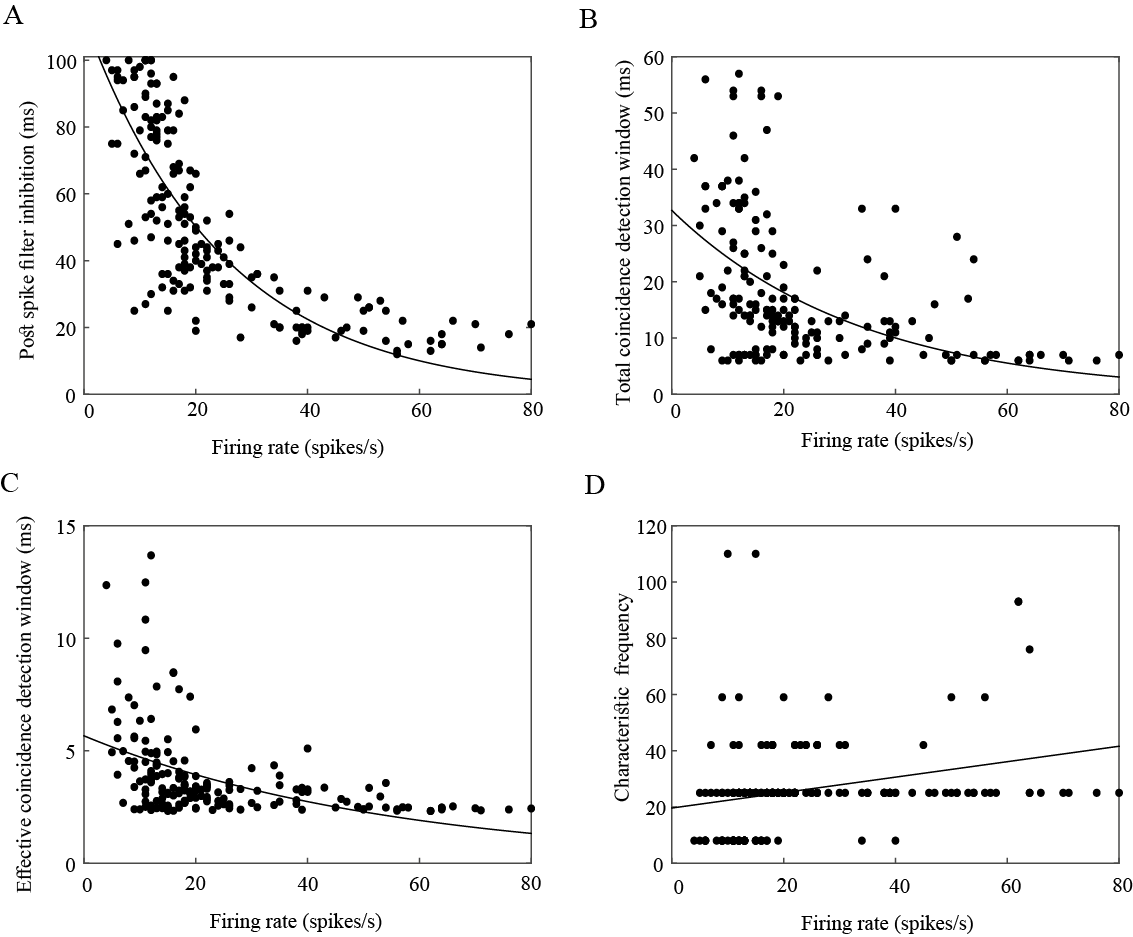


**Supplementary figure 1: The dependence of GLM parameters of cortical interneurons on the firing rate.** . In all cases, except *D*, the solid lines represent the exponential fitting functions. (A) Post spike filter inhibition, expressed as the latency to half height, as a function of the firing rate (R^2^=0.64, p>0.001). (B) Stimulus filter total coincidence detection window as a function of the firing rate (R^2^=0.22, p>0.001). (C) Stimulus filter effective coincidence detection window as a function of the firing rate (R^2^=0.19, p<0.001). (D) Stimulus filter characteristic frequency computed from the Fourier transform of the filter, as a function of the firing rate. Solid line represents the linear fitting function (R^2^=0.07, p>0.001)
